# Review of the mudflat varunid crab genus *Metaplax* (Crustacea, Brachyura, Varunidae) from East Asia and northern Vietnam

**DOI:** 10.3897/zookeys.877.38300

**Published:** 2019-10-02

**Authors:** Hsi-Te Shih, Jhih-Wei Hsu, Kingsley J.H. Wong, Ngan Kee Ng

**Affiliations:** 1 Department of Life Science, National Chung Hsing University, Taichung 402, Taiwan; 2 Research Center for Global Change Biology, National Chung Hsing University, Taichung 402, Taiwan; 3 Biodiversity Research Center, Academia Sinica, Taipei 115, Taiwan; 4 Department of Biological Sciences, National University of Singapore, 117543, Singapore

**Keywords:** COI, *Metaplax elegans*, *M. longipes*, *M. sheni*, *M. takahasii*, *M. tredecim*, mitochondrial cytochrome oxidase subunit I, morphology

## Abstract

Intertidal mudflat crabs of the genus *Metaplax* H. Milne Edwards, 1852 (Crustacea: Brachyura: Varunidae) from China, Taiwan, and northern Vietnam are taxonomically revised by morphological and molecular evidence. These crabs show sexual dimorphism and morphological variation of a considerable range in the infraorbital ridge, one of the primary features previously used for species identification. In this study, four species were identified from the region: *M. elegans* De Man, 1888; *M. longipes* Stimpson, 1858; *M. sheni* Gordon, 1930; and *M. tredecim* Tweedie, 1950. Based on the results of the morphological examination, and as confirmed by molecular evidence from mitochondrial cytochrome oxidase subunit I (COI), taxonomic confusion surrounding *M. longipes* was resolved, and *M. takahasii* Sakai, 1939, is considered a junior synonym of *M. longipes*. The geographical distribution of *Metaplax longipes* extends along the shores of China, north to Jiangsu, whereas the Southeast Asian *M. tredecim* was newly recorded from northern Vietnam and Hong Kong.

## Introduction

Indo-West Pacific varunid crabs of the genus *Metaplax* H. Milne Edwards, 1852 commonly inhabit sheltered shores with silty muddy substrate often near or under shades of mangroves in tropical and subtropical regions. While some studies have reported on various biological aspects of selected species (e.g., Macnae 1963; Beinlich and Polivka 1989; Chakraborty and Choudhury 1994), the phylogenetic position of this group within the Thoracotremata remains obscure due to limited taxon sampling (see Kitaura et al. 2002; Chen et al. 2019; Liu et al. 2019).

Species of the genus *Metaplax* all share a broad, subquadrate, somewhat depressed carapace, which is shallowly marked, broad fronted (approximately 1/3 carapace width), and has lateral margins bearing at most five distinct teeth; slender and elongated ambulatory legs are also shared. One of the frequently used morphological features for species identification remains the number of lobes and tubercles along the infraorbital ridge (Tesch 1918; Tweedie 1950; Dai et al. 1986; Dai and Yang 1991), which are sexually dimorphic as in many varunid groups. The infraorbital tubercles are reported to serve a stridulatory function, which engage with the ridge along the anterior margin of the chelipedal merus (Macnae 1963; Beinlich and Polivka 1989). *Metaplax* contains around 12 species (Ng et al. 2008; but see Naderloo 2011 on the identity of *M. indicus occidentalis* Pretzmann, 1971), with an overall distribution extending from the shores of the Persian Gulf, the Arabian Sea, the Bay of Bengal to Southeast and East Asia, and easternmost to western Taiwan. Six species, namely *M. elegans* De Man, 1888; *M. gocongensis* Davie & Nguyen, 2003; *M. longipes* Stimpson, 1858; *M. sheni* Gordon, 1930; *M. takahasii* Sakai, 1939; and *M. tredecim* Tweedie, 1950, have been recorded from East and Southeast Asia, with only *M. elegans* recorded in the eastern Indian Ocean as well (De Man 1888; Ng and Davie 2002; Dev Roy and Bhadra 2011).

In East Asia, the northern limit of this genus appears to be around Zhejiang, China (Dai et al. 1986; Chen 1991; Dai and Yang 1991). *Metaplax* is absent from the east coast of Taiwan, the Ryukyus, the main islands of Japan, and Korea (Sakai 1939, 1940, 1976). Five species were previously reported from the region, including *M. elegans* De Man, 1888, *M. longipes* Stimpson, 1858, *M. sheni* Gordon, 1930, *M. takahasii* Sakai, 1939, and *M. tredecim* Tweedie, 1950. Among these, *M. takahasii* has been considered a junior synonym of *M. longipes* (see Davie and Nguyen 2003), whereas tropical *M. tredecim* had been first listed as part of the fauna of the East China and South China seas by Yang et al. (2008) without any illustration or elaboration.

**Figure 1.**
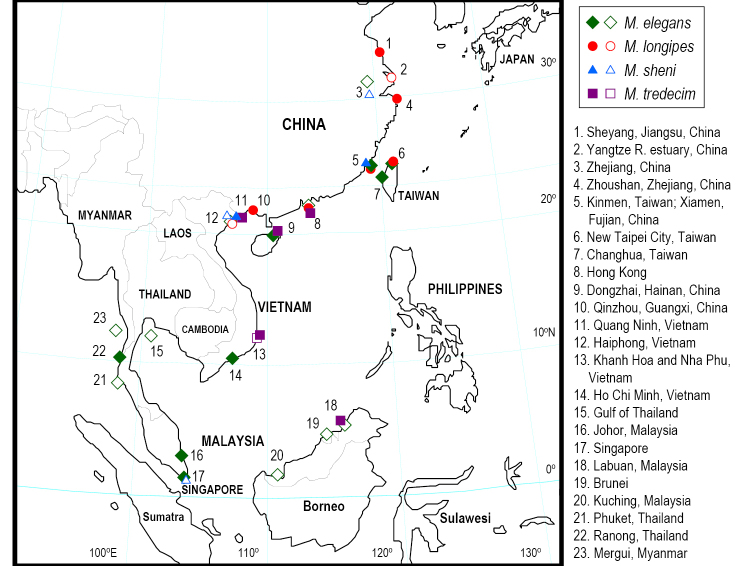
Collection sites (solid symbols) for species of the genus *Metaplax* used in this study: green rhombus for *M. elegans*; red circles for *M. longipes*; blue triangles for *M. sheni*; and purple squares for *M. tredecim*. Empty symbols mean the additional records from references (see synonym lists for each species).

In the present study, specimens of species of *Metaplax* were collected from various sites in East Asia (Fig. 1), their morphological features are examined and illustrated, and identifications are confirmed by molecular evidence from mitochondrial cytochrome oxidase subunit I (COI).

## Materials and methods

Specimens were collected from China, Taiwan, and Vietnam (Table 1, Fig. 1) and have been deposited in the Kanagawa Prefectural Museum of Natural History, Kanagawa, Japan (KPM-NH); the Zoological Collections of the Department of Life Science, National Chung Hsing University, Taichung, Taiwan (NCHUZOOL), and the Zoological Reference Collection of the Lee Kong Chian Natural History Museum, National University of Singapore, Singapore (ZRC). The abbreviation G1 is used for male first gonopods. Measurement is of the maximum carapace width (CW) in millimeters.

To understand whether the number of infraorbital tubercles and lobes of each species is related to the sex and body size, the numbers on both sides for each specimen were calculated, averaged, and plotted against CWs. Specimens used were 21 males (CW 7.7–15.9 mm) and 19 females (CW 6.3–13.2 mm) for *M. elegans*, 69 males (CW 6.8–26.6 mm) and 29 females (CW 7.9– 23.6 mm) for *M. longipes*, 7 males (CW 8.6–12.8 mm) for *M. sheni* (no female specimen), and 23 males (CW 12.8–22.7 mm) and 13 females (CW 10.3–23.4 mm) for *M. tredecim*.

Genomic DNA was isolated from the muscle tissue using extraction kits following Shih et al. (2016). A portion of the *COI* gene was amplified with PCR using the primers LCO1490 and HCO2198 (Folmer et al. 1994). PCR conditions for the above primers were denaturation for 50 s at 94 °C, annealing for 70 s at 45–47 °C, and extension for 60 s at 72 °C (40 cycles), followed by extension for 10 min at 72 °C. Sequences were obtained by automated sequencing (Applied Biosystems 3730), after verification with the complementary strand. Sequences of the different haplotypes have been deposited in the DNA Data Bank of Japan (DDBJ) (accession numbers are shown in Table 1). Outgroups were selected based on the phylogenetic tree of Kitaura et al. (2002: fig. 2), as follows: *Gaetice depressus* (De Haan, 1835); *Helice formosensis* Rathbun, 1931; *Hemigrapsus sanguineus* (De Haan, 1835); and *Varuna litterata* (Fabricius, 1798).

**Table 1.** The haplotypes and accession numbers (DNA Data Bank of Japan) of the COI gene of *Metaplax* specimens and outgroups from East Asia and northern Vietnam. For abbreviations of museums and universities, see Materials and methods.

Species	Locality	Sample size	Catalogue no. of NCHUZOOL (unless indicated)	Haplotype of COI	Access. no. of COI
*M. elegans*	Taiwan: Jhuwei, New Taipei City	1	15480	MXe1	LC498179
	Taiwan: Kinmen	1	15489	MXe2	LC498180
	Vietnam: Ho Chi Minh City	1	15499	MXe3	LC498181
	Singapore: Sungei Buloh	1	ZRC 1997.683	MXe4	LC498182
	Thailand: Ranong	1	15494	MXe4	LC498182
*M. longipes*	Taiwan: Danshuei, New Taipei City	1	NTOU	MXL2	LC498183
	Taiwan: Danshuei, New Taipei City	1	ZRC 1999.0708	MXL2	LC498183
	Taiwan: Kinmen	1	15460	MXL2	LC498183
	Taiwan: Kinmen	1	15462	MXL2	LC498183
	China: Zhoushan, Zhejiang	2	15466; 15465	MXL2	LC498183
	China: Xiamen, Fujian	1	15475	MXL2	LC498183
	China: Qinzhou, Guangxi	1	15449	MXL3	LC498185
	Hong Kong: Tung Chung	1	15450	MXL1	LC498184
*M. sheni*	Taiwan: Kinmen	1	15467	MXs1	LC498186
	China: Xiamen, Fujian	2	15465	MXs1, MXs2	LC498186, LC498187
	Vietnam: Dong Rui, Quang Ninh	1	15466	MXs1	LC498186
*M. tredecim*	Hong Kong: Nai Chung	1	15472	MXt1	LC498188
	Vietnam: Dong Rui, Quang Ninh	1	15477	MXt2	LC498189
	Vietnam: Nha Trang	1	15498	MXt3	LC498190
	Malaysia: Labuan	1	15475	MXt4	LC498191
**Total**		**22**			
**Outgroups**					
*Gaetice depressus*	Taiwan: Keelung		15544		LC498192
*Helice formosensis*	Taiwan: Shengang, Changhua		13083		AB334543
*Hemigrapsus sanguineus*	Taiwan: Yongsing, New Taipei City		15545		LC498193
*Varuna litterata*	Taiwan: Kenting, Pingtung		14816		LC498194

**Figure 2.**
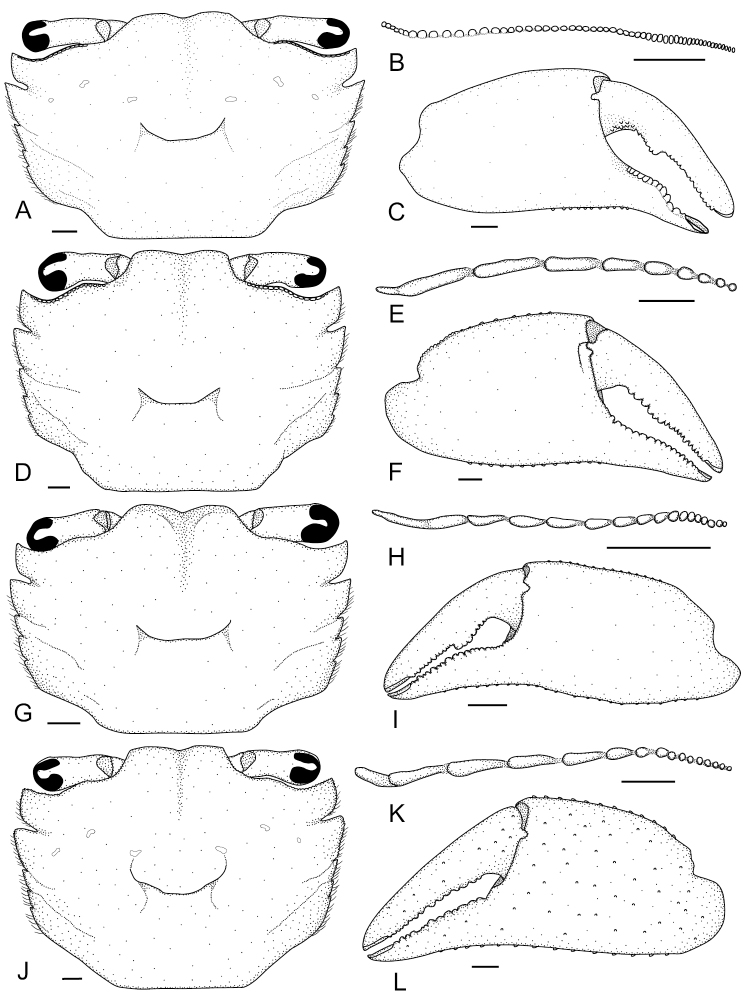
*Metaplax elegans* De Man, 1888 (**A–C**NCHUZOOL 15496, male, 12.7 mm), *M. longipes* Stimpson, 1858 (**D–F**ZRC 2019.0581, male, 14.9 mm), *M. sheni* Gordon, 1930 (**G–I**NCHUZOOL 15466, male, 9.9 mm), and *M. tredecim* Tweedie, 1950 (**J–L** paratype, ZRC 1964.7.14.4-18, 16.2 mm). **A, D, G, J, A** carapace **B, E, H, K** left infraorbital ridge **C, F, I, L** outer view of right cheliped. Scale bars: 1.0 mm.

The best-fitting model of sequence evolution was determined by PartitionFinder (ver. 2.1.1; Lanfear et al. 2017) and selected by the Bayesian information criterion (BIC). The obtained best model (GTR + I + G) was subsequently used for a Bayesian inference (BI) analysis. BI analysis was performed with MrBayes (ver. 3.2.3, Ronquist et al. 2012). Phylogenetic analysis was performed with four chains for 10 million generations and four independent runs, with trees sampled every 1000 generations. The convergence of chains was determined by the average standard deviation of split frequency values below the recommended 0.01 (Ronquist et al. 2019), and the first 1000 trees were discarded as the burnin accordingly. Maximum likelihood (ML) analysis was conducted using RAxML (vers. 7.2.6, Stamatakis 2006). The model GTR + G (i.e. GTRGAMMA) was used with 100 runs and finding the best ML tree by comparing the likelihood scores. The robustness of the ML tree was evaluated by 1000 bootstrap pseudoreplicates under the model GTRGAMMA. Base pair (bp) differences and pairwise estimates of Kimura 2-parameter (K2P) distances (Kimura 1980) for genetic diversities between specimens were calculated with MEGA (ver. 10.0.5, Kumar et al. 2018).

## Results

### Systematics

**Family Varunidae H. Milne Edwards, 1853**

**Genus *Metaplax* H. Milne Edwards, 1852**

#### Metaplax elegans

Taxon classificationAnimaliaDecapodaVarunidae

De Man, 1888

AF9022BC-3B84-59F5-86CE-B87FC3E1DB85

**Figure 3.**
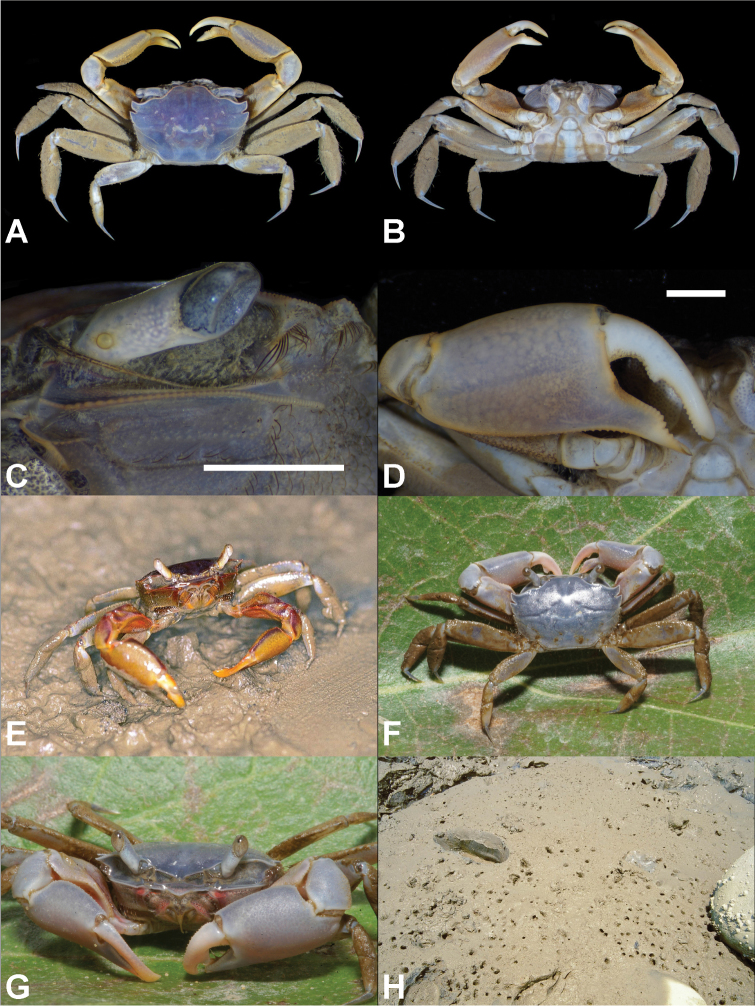
*Metaplax elegans* De Man, 1888. **A** Dorsal view **B** ventral view **C** left infraorbital ridge **D** right cheliped **A–D** male (CW 12.7 mm; NCHUZOOL 15496; Dongshih, Chiayi County, Taiwan) **E, F** live coloration **E** photo taken in Gaomei, Taichung, Taiwan (specimen not collected) **F–G** male (CW 13.1 mm; NCHUZOOL 15489; Kinmen, Taiwan) **G** the typical habitat (Gaomei, Taichung, Taiwan). Scale bars: 2.0 mm.

Figures 2A–C , 3 , 7A–D

Metaplax elegans De Man, 1888: 164, pl. 11(4–6) (type locality: Mergui, Myanmar); Alcock 1900: 434 (East India: Godavari Delta; Myanmar: Mergui); Gordon 1931: 528 (Hong Kong); Rathbun 1931: 100 (China: Fujian; Guangdong); Tweedie 1936: 69 (Malaysia: Selangor; Singapore); Shen 1940a: 74, 95 (China: Zhejiang; Fujian); Shen 1940b: 236 (Hong Kong); Tweedie 1950: 353 (Malaysia: Labuan; Kuching); Macnae 1963: 104, 180 (list); Dai et al. 1986: 509, fig. 289 (1–2), pl. 72(5) (China: Guangdong); Fukui et al. 1989: 230, fig. 25 (W Taiwan); Dai and Yang 1991: 557, fig. 289 (1–2), pl. 72(5) (China: Guangdong); J.-T. Shih et al. 1991: 126 (Taiwan: New Taipei City); Davie 1992: 352, pl. 2B (Hong Kong); Choy and Booth 1994: 243 (Brunei); Huang 1994: 598 (list; China); Tan and Ng 1994: 82 (Singapore; Malaysia); Kuo 1995: 31, 82, 97, 191, 4 unnumbered figs (W Taiwan); Wang and Liu 1996a: 128, figs 171–172 (W Taiwan); Wang and Liu 1996b: 103–104, 2 unnumbered figs (W Taiwan); Fransen et al. 1997: 125 (syntype; Mergui, Myanmar); Ho and Hung 1997: 108–109, 1 unnumbered fig.; Kosuge et al. 1997: 182 (Vietnam: Haiphong); Jeng et al. 1998: 68, 3 unnumbered figs (Taiwan: Taichung); Wang and Liu 1998a: 128, figs 171–172 (W Taiwan); Wang and Liu 1998b: 142, 2 unnumbered figs (W Taiwan); Lee and Leung 1999: 68, pl. 11 (Hong Kong); Ng and Sivasothi 1999: 73, 2 unnumbered figs (Singapore); Jeng and Wang 2000: 38, 2 unnumbered figs (Taiwan: Taichung); Lee and Tung 2000: 70 (list); Dev Roy and Bhadra 2011: 36 (list; E India); Lee 2001: 114, 3 unnumbered figs (W Taiwan); Ng et al. 2001: 45 (list; Taiwan); Kitaura et al. 2002: 684 (Vietnam: Haiphong); Ng and Davie 2002: 379 (Thailand: Phuket); Wang and Liu 2003: 128, figs 171–172 (W Taiwan); Naiyanetr 2007: 112 (list: Gulf of Thailand); Dev Roy 2008: 135 (list; India); Huang 2008: 668 (list; China); Ng et al. 2008: 226 (list); Yang et al. 2008: 803 (list; East and South China seas); Rath and Dev Roy 2008: 72, pl. 4(5) (NE India); Liu and Wang 2010: 72, 3 unnumbered figs (W Taiwan); Lee et al. 2013: 108, 2 unnumbered figs (Taiwan: Tainan); Ng et al. 2017: 110 (list; Taiwan).

##### Materials examined.

**Taiwan**: 8 ♂♂ (6.1–14.0 mm), 4 ♀♀ (8.0–13.2 mm) (NCHUZOOL 15479), Danshuei River mangroves, New Taipei City, coll. P.-Y. Hsu and J.-W. Hsu, 24 Mar. 2017; 4 ♂♂ (11.8–15.9 mm), 4 ♀♀ (14.1–15.3 mm) (NCHUZOOL 15480), Jhuwei, New Taipei City, 4 Oct. 1995; 1 ♂ (12.1 mm) (NCHUZOOL 15481), Sinfeng, Hsinchu, 15 Jan. 2014; 2 ♂♂ (8.4–10.5 mm), 5 ♀♀ (6.3–12.1 mm) (NCHUZOOL 15482), Siangshan, Hsinchu, 11 Aug. 2008; 1 ♂ (13.2 mm), 5 ♀♀ (8.7–9.7 mm) (NCHUZOOL 15483), Siangshan, Hsinchu, 13 Mar. 2008; 4 ♂♂ (10.1–11.3 mm), 1 ♀ (11.7 mm) (NCHUZOOL 15484), Haishangu, Hsinchu, 27 Aug. 2013; 1 ♂ (10.8 mm), 3 ♀♀ (8.8–11.6 mm) (NCHUZOOL 15485), Wufu Bridge, Miaoli, 2 Dec. 2015; 4 ♂♂ (12.0–15.1 mm) (NCHUZOOL 15486), Fangyuan, Changhua, 2014; 4 ♂♂ (10.0–12.2 mm), 6 ♀♀ (8.1–12.1 mm) (NCHUZOOL 15487), Yuliao R. estuary, Changhua, coll. J.-W. Hsu et al., 16 Jan. 2017; 4 ♂♂ (5.3–10.6 mm), 3 ♀♀ (10.2–12.9 mm) (NCHUZOOL 15488), area between Yunlin and Chiayi, coll. K.-C. Li and C.-T. Wang, 25 Aug. 2003; 1 ♂ (12.7 mm) (NCHUZOOL 15496), Dongshih, Chiayi County, coll. P.-Y. Hsu, 24 Jan. 2017; 4 ♂♂ (11.5–13.1 mm), 3 ♀♀ (6.7–10.7 mm) (NCHUZOOL 15489), Cihhu, Kinmen, coll. H.-T. Shih and P.-Y. Hsu, 29 June 2018. **China**: 3 ♀♀ (14.0–16.8 mm) (NCHUZOOL 15495), Dongzhai Harbor, Hainan, 23 June 2004; 3 ♀♀ (13.9–16.6 mm) (NCHUZOOL 15457), Dongzhai Harbor, Hainan, 23 June 2004. **Vietnam**: Ho Chi Minh: 8 ♂♂ (5.8–16.5 mm), 9 ♀♀ (6.5–13.7 mm) (NCHUZOOL 15490), Rung Sac, Long Hoa, 12 Oct. 2017; 1 ♂ (18.2 mm), 1 ♀ (12.7 mm) (NCHUZOOL 15491), TT. Can Thanh mangroves, Can Gio, 13 Oct. 2017; 5 ♂♂ (16.0–17.6 mm) (NCHUZOOL 15499), TT. Can Thanh mangroves, Can Gio, 13 Oct. 2017. **Malaysia**: 7 ♂♂ (7.7–12.6 mm), 14 ♀♀ (8.9–12.0 mm) (NCHUZOOL 15492), Mersing, Johor, 19 July 2010. **Singapore**: 1 specimen (not examined, only for DNA study; ZRC 1997.683), Sungei Buloh, 1996; 2 ♂♂ (9.4–11.0 mm), 1 ♀ (6.4 mm) (NCHUZOOL 15493), Lim Chu Kang, 4 Mar. 2012. **Thailand**: 1 ♀ (10.71 mm) (NCHUZOOL 15494), Ranong mangroves, 27 May 2012.

##### Diagnosis.

Carapace (Figs 2A, 3A, F) trapezoidal, 1.45 times as broad as long (*N* = 127, SD = 0.06), longitudinally convex, broadest between lateral teeth 2 (exorbital angle included), surface sparsely but regularly furnished with short, stiff setae; front broad, divided into two broad lobes, medially concave; lateral margins markedly converging posteriorly, interrupted by four notches, delineating five teeth (including exorbital angle), exorbital angle most distinct, posterior two indistinct; posterolateral facet faintly defined, decorated by two oblique granular ridges. Infraorbital ridge (Figs 2B, 3C) distinctly sexual dimorphic, males with 47–61 tubercles, medial seven closely set, almost fusing, lateral ca. 20 tubercles slightly vertically elongated; females with 33–42 isomorphic tubercles. Chelipeds (Figs 2C, 3D) symmetrical, in males more elongated and robust, palm 2.2 times as long as broad, length of palm approximately 1.8 times longer than dactyl (*N* = 20), pollex of chela with low sinuous tooth along cutting margin, dactylus with distinct triangular molar. Ambulatory legs elongated, meri broad, merus of P4 distally armed with several short spines on anterior margin; anterior margins of all ambulatory legs fringed with setal patches. G1 (Fig. 7A–D) long, slender, distal process triangular, distinctly curved outward.

##### Distribution.

The Bay of Bengal, Southeast and East Asia: China (Zhejiang; Fujian; Guangdong; Hainan), western Taiwan (including Kinmen), Vietnam, Malaysia (Selangor; Sarawak; Labuan), Singapore, Brunei, Thailand, Myanmar (Mergui), and eastern India (Tamil Nadu, Godavari Delta) (Fig. 1).

##### Habitat.

At Wazihwei Wetland, estuaries of Danshuei River, northwestern Taiwan, this species is found along shores with substantial freshwater influence, on banks with substrates plastic-muddy, somewhat distant from mangrove stands.

##### Remarks.

According to previous descriptions (De Man 1888, Dai et al. 1986, Dai and Yang 1991), the infraorbital ridges of *M. elegans* bear 50–60 tubercles in males and 35–40 tubercles in females. In the present study of 21 males and 19 females, CW ranging from 7.7 to 15.9 mm, and the range of variation is slightly wider than previously reported, being 47–61 in males and 33–42 isomorphic tubercles in females (Table 2, Fig. 8).

**Table 2.** Comparison of characters among four species of *Metaplax* from East Asia and northern Vietnam.

Characters	*M. elegans*	*M. longipes*	*M. sheni*	*M. tredecim*
lateral margin	five teeth	five teeth	five teeth	five teeth
infraorbital ridge	46–61 tubercles in males (lateral 20 vertically elongated); 33–42 isomorphic tubercles in females	7–13 tubercles in males (mesial ones broad, and gradually decreasing in size); 14–22 isomorphic tubercles in females	16–20 tubercles in males (mesial ones broad, decreasing in size laterally; mesial-most one more than twice the breadth of the next)	13–20 tubercles in males (mesial ones broad and decreasing in size, lateral 4–5 roughly same size); 20–27 isomorphic tubercles in females
cheliped	palm 2.2 times as long as broad, total length of palm nearly 1.8 times than length of dactyl, cutting edge of dactylus with distinct large teeth	palm 2.3 times as long as broad, length of palm nearly 1.3 times than length of dactyl, cutting edge of dactylus with low triangular molar, pollex unarmed	markedly elongated, palm 2.8 times as long as broad, length of palm nearly 2.0 times than length of dactylus, cutting edge of dactylus with triangular molar	palm 2.3 times as long as broad, length of palm nearly 1.6 times than length of dactyl, cutting edge of both fingers unarmed
ambulatory legs	short, broad	long, slender	long, slender	long, slender

#### Metaplax longipes

Taxon classificationAnimaliaDecapodaVarunidae

Stimpson, 1858

33361C4E-9490-570D-A234-E795A69983A1

**Figure 4.**
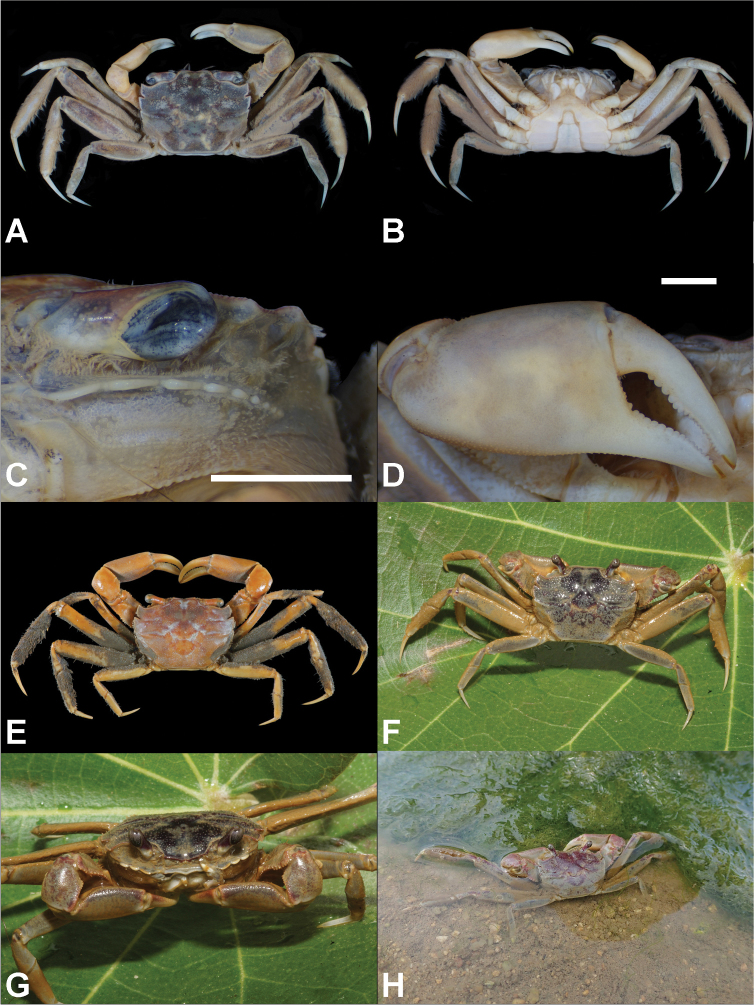
*Metaplax longipes* Stimpson, 1858. **A, E** Dorsal view **B** ventral view **C** left infraorbital ridge **D** right cheliped. **A–D** Male (CW 15.4 mm; ZRC 2019.0581; Hong Kong) **E** male (CW 24.1; ZRC 1999.0708; Danshuei, northwestern Taiwan) **F–H** live coloration **F–G** male (CW 20.6 mm; NCHUZOOL 15553; Lieyu, Kinmen, Taiwan) **H** male (CW 23.7 mm; NCHUZOOL 15501; Hong Kong). Scale bars: 2.0 mm.

Figures 2D–F , 4 , 7E–H

Metaplax longipes Stimpson, 1858: 97 (type locality: Hong Kong); Koelbel 1897: 711, pl. 1(5–6) (Hong Kong); Stimpson 1907: 99 (Hong Kong); Tesch 1918: 116 (key; no new specimens); Gee 1926: 164 (China: “Chin Bey”); Gordon 1931: 528 (Hong Kong; China: Amoy (= Xiamen), Fujian); Shen 1940a: 74, 95 (China: Zhejiang; Fujian); Shen 1940b: 236 (Hong Kong); Shen and Dai 1964: 133, 1 unnumbered fig. (China: Zhejiang; Fujian; Guangdong); Dai et al. 1986: 508, fig. 288 (1–2), pl. 72(3) (China: Zhejiang; Fujian; Guangdong) (part); Chen 1991: 441, fig. 416; Dai and Yang 1991: 556, fig. 288 (1–2), pl. 72(3) (China: Zhejiang; Fujian; Guangdong) (part); Davie 1992: 352 (key); Huang 1994: 598 (list; China); Wang and Liu 1996c: 227 (list); Lee 2001: 115, 3 unnumbered figs (W Taiwan); Ng et al. 2001: 54 (list; Taiwan); Davie and Nguyen 2003: 384 (no specimen examined); Wang 2003: 111, 1 unnumbered fig. (Taiwan: Kinmen); Liu and He 2007: 167: 1 unnumbered fig. (China: Yangtze R. estuary); So and Lui 2007: 36, 3 unnumbered figs (Hong Kong); Huang 2008: 668 (list; China); Ng et al. 2008: 226 (list); Yang et al. 2008: 803 (list; East and South China seas); Ng et al. 2017: 110 (list; Taiwan).
Metaplax takahasii Sakai, 1939: 698, text-fig. 127 (type locality: Tansui (= Danshuei), Taiwan); Sakai 1940: 58 (list; Japan; Taiwan); Lin 1949: 31 (list; Taiwan); Fukui et al. 1989: 230, fig. 24 (Taiwan: New Taipei City); Dai and Yang 1991: 556, fig. 288 (3–4), pl. 72(4) (China: Fujian; Guangdong); J.-T. Shih et al. 1991: 126 (Taiwan: New Taipei City); Davie 1992: 352, pl. 2A (Hong Kong); Lee and Leung 1999: 69 (Hong Kong).
Metaplax takahashii : Horikawa 1940: 30 (list; Taiwan); Sakai 1976: 673, text-fig. 371 (Taiwan: Danshuei); Dai et al. 1986: 508, fig. 288 (3–4), pl. 72(4) (China: Fujian; Guangdong); Huang 1994: 598 (list; China); Kosuge et al. 1997: 182 (Vietnam: Haiphong); Muraoka 1998: 54 (Danshuei R., Taiwan); Ng et al. 2001: 46 (list; Taiwan); Kitaura et al. 2002: 684 (Vietnam: Haiphong); Davie and Nguyen 2003: 384 (no new specimens); Ng et al. 2017: 110 (list; Taiwan); Huang 2008: 668 (list; China); Ng et al. 2008: 226 (list; Taiwan).
? Metaplax longipes: Naiyanetr 2007: 112 (list: Gulf of Thailand).
Not Metaplax longipes: Davie 1992: 352 (key) (= Metaplax tredecim Tweedie, 1950).
Not Metaplax longipes: Chertoprud et al. 2012: 276, pl. 47F (Nha Phu, southeastern Vietnam) (= Metaplax tredecim Tweedie, 1950).

##### Materials examined.

**China**: 2 ♂♂ (20.7–22.7 mm) (NCHUZOOL 15443), Sheyang, Jiangsu, coll. W.-R. Lin, 24 Aug. 2015; 3 ♂♂ (11.3–22.3 mm), 1 ♀ (21.6 mm) (NCHUZOOL 15444), Mamu, Zhoushan, Zhejiang, 26 July 2018; 6 ♂♂ (15.9–26.6 mm), 3 ♀♀ (18.0–19.15 mm) (NCHUZOOL 15446), Mamu, Zhoushan, Zhejiang, Sep. 2018; 2 ♂♂ (13.9–15.6 mm), 5 ♀♀ (13.6–23.6) (NCHUZOOL 15447), Mamu, Zhoushan, Zhejiang, 26 July 2018; 1 ♀ (16.8 mm) (NCHUZOOL 15445), Buqiangwan, Zhoushan, Zhejiang, 26 July 2018; 2 ♂♂ (12.0–12.1 mm), 4 ♀♀ (9.8–18.6 mm) (NCHUZOOL 15448), Liuwudian, Xiamen, Fujian, 31 July 2018; 1 ♂ (19.2 mm) (NCHUZOOL 15449), Qinzhou, Guangxi, 10 May 2009. **Hong Kong**: 1 ♂ (18.3 mm) (NCHUZOOL 15451), Tung Chung, coll. K. J. H. Wong, 21 Mar. 2009; 1 ♂ (15.4 mm) (ZRC 2019.0581), 4 ♂♂ (11.2–16.7 mm) (NCHUZOOL 15450), Tung Chung, coll. K. J. H. Wong, 9 Apr. 2016; 1 ♂ (9.3 mm), 2 ♀♀ (8.6–9.8 mm) (NCHUZOOL 15452), Tung Chung, coll. K. J. H. Wong, 11 July 2015; 15 ♂♂ (6.6–12.7 mm), Tung Chung, coll. K. J. H. Wong, 17 July 2015; 4 ♂♂ (6.0–8.8 mm) (NCHUZOOL 15455), Tung Chung, coll. K. J. H. Wong, 18 July 2011; 3 ♂♂ (17.7–23.7 mm), 1 ♀ (13.3 mm), Tung Chung, coll. K. J. H. Wong, 22 Apr. 2019; 3 ♂♂ (6.8–8.6 mm), 3 ♀♀ (10.0–14.7 mm) (NCHUZOOL 15503), Tung Chung, coll. H.-T. Shih and K. J. H. Wong, 2 June 2019; 1 ♂ (17.6 mm) (NCHUZOOL 15502), Lantau Island, 2 June 2019; 2 ♂♂ (9.9–22.3 mm); 1 ♂ (24.8 mm), 1 ovig. ♀ (19.0 mm) (ZRC 2019.0542), ca. 22.495486N, 114.029947E, mudflats at mangroves, Mai Po Nature Reserve, coll. K. J. H. Wong, 24 May 2019. **Macao**: 1 ♀ (17.5 mm) (NCHUZOOL 15454), Coloane, coll. K. J. H. Wong, 3 July 2015. **Taiwan**: 1 ♀ (17.7 mm) (KPM-NH 0107076), Danshuei, New Taipei City, coll. S. Takahashi (?), 1933 (?); 1 ♀ (14.7 mm) (NTOU), Danshuei, New Taipei City, 25 May 1984; 1 ♀ (14.4 mm) (NTOU), Danshuei, New Taipei City, coll. L.-H. Hsieh, 7 May 1992; 2 ♂♂ (10.6–20.0 mm) (ASIZ), Danshuei River mangroves, New Taipei City, 17 Mar. 1986; 1 ♂ (24.1 mm) (ZRC 1999.0708), Danshuei, New Taipei City, 8 July 1999; 1 ♂ (21.8 mm) (ZRC 1999.0708), Danshuei, New Taipei City, 8 July 1999; 3 ♂♂ (6.9–17.4 mm), 2 ♀♀ (7.9–10.3 mm) (NCHUZOOL 15458), Wujiang R. estuary, Kinmen, 6 Mar. 2008; 1 ♂ (19.1 mm), 1 ♀ (16.9 mm) (NCHUZOOL 15459), Wujiang R. estuary, Kinmen, 16 Aug. 2011; 7 ♂♂ (7.4–14.6 mm), 1 ♀ (9.3 mm) (NCHUZOOL 15460), Wujiang R. estuary, Kinmen, coll. H.-T. Shih and P.-Y. Hsu, 29 June 2018; 2 ♂♂ (7.0–7.1 mm) (NCHUZOOL 15461), Cihhu, Kinmen, coll. H.-T. Shih and P.-Y. Hsu, 29 June 2018; 1 ♀ (19.9 mm) (NCHUZOOL 15462), Mashan, Kinmen, 17 Aug. 2011; 1 ♀ (21.1 mm) (NCHUZOOL 1551), 1 ♀ (5.9 mm) (NCHUZOOL 15552), 1 ♂ (20.6 mm), 1 ♀ (17.5 mm) (NCHUZOOL 15553), 1 ♀ (20.1 mm) (NCHUZOOL 15554), Lieyu, Kinmen, coll. H.-T. Shih and P.-Y. Hsu, 28 June, 2018; 1 ♂ (18.1 mm) (NCHUZOOL 15463), Cingshuei, Matsu, coll. J.-H. Li, 9 July 2005; 1 ♂ (20.0 mm) (NCHUZOOL 15464), Cingshuei, Matsu, coll. J.-H. Li, 9 July 2005.

##### Diagnosis.

Carapace (Figs 2D, 4A, E, F) subquadrate, 1.36 times broader than long (*N* = 98, SD = 0.05), mildly convex longitudinally and laterally, region faintly defined; front medially slightly concave; lateral margin nearly parallel, interrupted by four notches (cutting into five teeth), anterior two lateral teeth pronounced, posterior two very indistinct; posterior facet depressed, decorated by two oblique granular ridges, anterior one extended from second notch. Infraorbital ridge (Figs 2E, 4C) marked sexually dimorphic: males with 7–13 lobes and tubercles, medial 2 broad, decreasing in breadth laterally, innermost four or five decreasing in size, lateral ones small, isomorphic; females with 16–22 small isomorphic tubercles. Chelipeds (Figs 2F, 4D) symmetrical, robust, palm 2.3 times as long as broad, length of palm approximately 1.3 times length of dactyl (*N* = 16), merus denticulate along anterior and posterior margins; chelae surface smooth, pollex and dactylus unarmed of pronounced molars along cutting edge. Ambulatory legs slender, elongated, meri unarmed along anterior margin, proximal half of meri, and propodi of all furnished with setal mats. G1 (Fig. 7E–H) elongated, relatively stout, almost straight.

##### Distribution.

Western Taiwan (including Matsu and Kinmen), China (Jiangsu; Zhejiang; Fujian; Guangdong; Guangxi), and northern Vietnam (Haiphong) (Fig. 1). The record in the Gulf of Thailand (Naiyanetr 2007: 112) requires further verification.

##### Habitat.

At Tung Chung Wetland, Hong Kong, where numerous specimens were collected, the habitat of this species is composed of muddy substrates and substantial freshwater influences. Considerable numbers flourish under fringes of mangrove stands, as well as the adjacent more open mudflats.

##### Remarks.

The identity of *Metaplax longipes* had long remained unclear since the publication of *M. takahasii* Sakai, 1939. The confusion between the two nominal species was mainly caused by two crucial morphological features used for species identification: the number of tubercles and lobes along the male infraorbital ridge, and the number of teeth on the lateral margin of the carapace.

Originally described from Hong Kong by Stimpson (1858, 1907), type material(s) of *M. longipes* was destroyed in the Great Chicago Fire in 1871 (Evans 1967). Illustrations based on material from Hong Kong were eventually presented by Koelbel (1897: pl. 1(5–6)), and further records from Hong Kong and elsewhere in South China include those by Gee (1926), Gordon (1931), Shen (1940a, b) and Shen and Dai (1964). Morphology of the infraorbital ridge in males serves as a good taxonomic character (Tesch 1918); delimitations provided in various work under the name *M. longipes* range from 7 to 10: “seven-lobed” (Stimpson 1907), “fogak száma összesen tehát kilencz” (= total number of nine teeth; Koelbel 1897), “number of lobules or teeth … 7–9” (Tesch 1918, Gordon 1931), and “9 to 10 tubercles” (Shen and Dai 1964).

Interpretations of *M. longipes* by Dai et al. (1986) and Dai and Yang (1991) brought much confusion. These authors illustrated two forms of infraorbital ridges based on specimens from South China (Guangdong to Zhejiang), one bearing 17 lobes and tubercles, and the other bearing nine (fig. 288(1) in Dai et al. 1986 and Dai and Yang 1991). It appeared very likely that their material was composite (also see Remarks under *M. tredecim*). Anyhow, this “shift” in the number of tubercles was subsequently followed by various authors: “with 15–17 lobules and teeth” (Davie 1992) and “about 9–17 tubercles” (Lee and Leung 1999). Reflecting this confused situation, the dichotomous key to the *Metaplax* species provided by Davie (1992: 352), which differentiated “*M. longipes*” (15 to 17 lobules and teeth) from “*M. takahasii*” (9 teeth), was problematic. Following diagnoses given by Stimpson (1858, 1907), and authors such as Gordon (1931) and Shen and Dai (1964), only those with around 9 lobes or tubercles, should be considered as the true *M. longipes*.

Without accessing any material of *M. longipes* from South China, Sakai (1939) described a similar form named *M. takahasii* based on one male specimen from Tansui (= Danshuei), northwestern Taiwan, after the naturalist and collector, Sadae Takahashi (or Sadae Takahasi in another translation). The species was subsequently reported elsewhere in China, including Guangdong and Fujian (Dai et al. 1986; Dai and Yang 1991) and Hong Kong (Davie 1992). Regarding the correct spelling of the species epithet, “*takahasii*” (original as in Sakai 1939), instead of “*takahashii*” as in Sakai (1976), should be maintained (ICZN 1999: Article 32.2).

Nevertheless, *M. takahasii* was described with an infraorbital ridge composed of 8 tubercles and the lateral margin of the carapace cut into five teeth. Considering the original descriptions of *M. longipes* and *M. takahasii* (Stimpson 1907 and Sakai 1939, respectively), holotypes of the two (CW 15.5 mm and 14.2 mm, respectively) differ by the numbers of lateral carapace teeth (four vs. five) of the carapace and the infraorbital lobes and tubercles (seven vs. eight). The number of infraorbital tubercles of both forms overlap might be explained by variation between intraspecific individuals (see “Note on the number of infraorbital tubercles and lobes”; Fig. 8), whereas the posterior-most notch along the lateral margin, however, can be very indistinct and often obscured by a layer of sediment-laden setae and easily omitted (Davie and Nguyen 2003; see Remarks under *M. tredecim* below). This led Davie and Nguyen (2003: 384) to the view that *M. longipes* is “almost certain(ly) … a senior synonym of *M. takahashii*”. In enumerating Chinese species of *Metaplax*, Yang et al. (2008: 803), probably following Davie and Nguyen’s (2003) suggestion, listed *M. takahasii* as a junior synonym of *M. longipes* without further elaboration. In our material referred to *M. longipes*, the number of infraorbital tubercles and lobes varies from 7–13 for males and 14–22 in females (Table 2; Fig. 8)

In the present study, we compared specimens from Hong Kong (identified as *M. longipes*) and various lots from Taiwan main island (originally labeled as *M. takahasii*: see Materials examined above) with morphological and molecular approaches. As noted by Davie and Nguyen (2003; also see above), the number of notches (hence teeth) on the lateral margin of the carapace is easily underestimated unless the surface is carefully denuded. This aspect is well-illustrated in the case of *M. tredecim* (as discussed below), and also between specimens of *M. longipes* from Hong Kong (Fig. 4A) and “*M. takahasii*” from Danshuei, Taiwan (Fig. 4E), the two being identical. Molecular analyses also support only one clade of specimens from various localities of China and Taiwan (Table 1; Fig. 9).

#### Metaplax sheni

Taxon classificationAnimaliaDecapodaVarunidae

Gordon, 1930

403949D3-DF38-52E2-83A9-8ED25C641F0A

**Figure 5.**
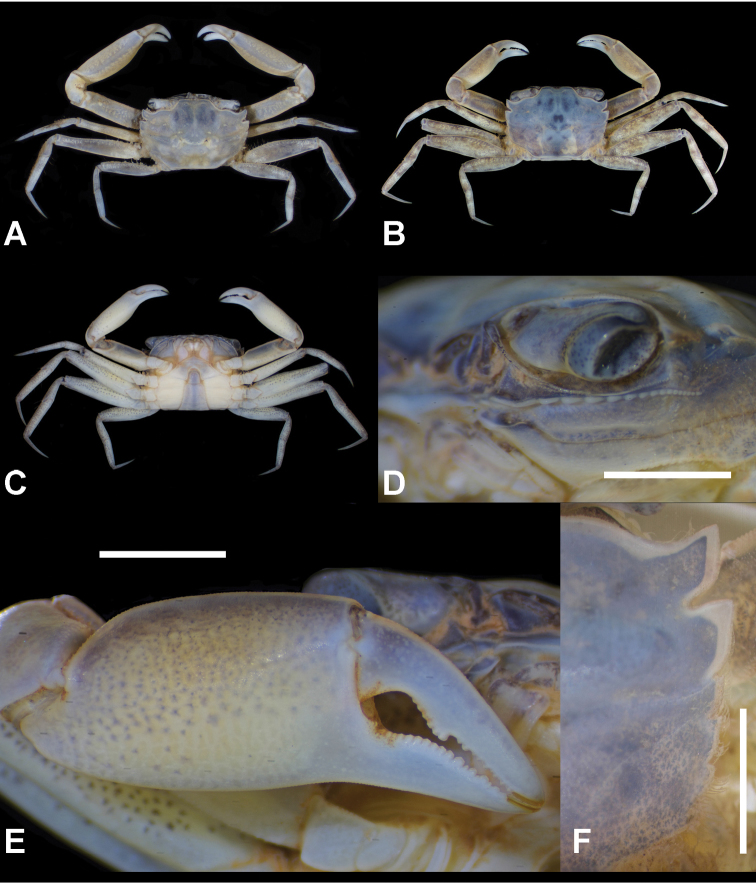
*Metaplax sheni* Gordon, 1930. **A, B** dorsal view **C** ventral view **D** left infraorbital ridge **E** right cheliped **F** right side of carapace showing the five teeth of lateral margin. **A, C** Male (CW 12.8 mm; NCHUZOOL 15465; Xiamen, Fujian, China) **B, D–F** male (CW 9.9 mm; NCHUZOOL 15466; Dong Rui, Quang Ninh, Vietnam). Scale bars: 2.0 mm.

Figures 2G–I , 5 , 7I–L

Metaplax sheni Gordon, 1930: 525 (type locality: Amoy (= Xiamen), Fujian, China); Gordon 1931: 553, figs 31–32 (China: Xiamen, Fujian); Tweedie 1936: 69, fig. 15(5) (Singapore); Shen 1940a: 74, 95 (China: Zhejiang; Fujian); Shen and Dai 1964: 133, 1 unnumbered fig. (China: Fujian); Dai et al. 1986: 509, fig. 289 (3–4), pl. 72 (6) (China: Fujian); Chen 1991: 441, fig. 415 (China: Zhejiang); Dai and Yang 1991: 558, fig. 289 (3–4), pl. 72(6) (China: Fujian); Huang 1994: 598 (list; China); Tan and Ng 1994: 82 (Singapore); Kosuge et al. 1997: 182 (Vietnam: Haiphong); Kitaura et al. 2002: 684 (Vietnam: Haiphong); Davie and Nguyen 2003: 383 (Malaysia: Johor; Singapore); Huang 2008: 668 (list; China); Ng et al. 2008: 226 (list); Yang et al. 2008: 803 (list; China: Fujian).
Metaplax indica : Rathbun 1931: 100 (China: Fujian); Shen 1940a: 74, 95 (list; South China). (not M. indica H. Milne Edwards, 1852)

##### Materials examined.

**China**: 5 ♂♂ (8.6–12.8 mm) (NCHUZOOL 15465), Wuyuanwan, Xiamen, Fujian, coll. H.-T. Shih et al., 1 Aug. 2018. **Taiwan**: 1 ♂ (9.9 mm) (NCHUZOOL 15467), Kinmen. **Vietnam**: 1 ♂ (9.9 mm) (NCHUZOOL 15466), Dong Rui, Quang Ninh, coll. H.-T. Shih et al., 29 May 2016.

##### Diagnosis.

Carapace (Figs 2G, 5A, B) subquadrate, 1.45 times broader than long (*N* = 7, SD = 0.04), longitudinally convex, regions faintly defined; frontal margin sinuous, medially noticeably concave; lateral margin markedly converging posteriorly, furnished with a row of soft setae, interrupted by four notches, cutting into five teeth, anterior two marked, triangular, last two weak, indistinct. Infraorbital ridge (Figs 2H, 5D) of males with 16–20 lobes and tubercles, innermost tubercle more than twice as broad as the adjacent, followed by six broad tubercles, decreasing in size. Chelipeds (Figs 2I, 5E) of males subequal, markedly elongated, palm 2.8 times as long as broad, length of palm approximately 2 times longer than length of dactyl (*N* = 6), merus and palm subequal in length; both fingers about half-length of palm, deflexed; cutting edges of pollex with low, serrated lobe, dactylus bearing distinct triangular molar. Ambulatory legs slender, elongated, anterior margins of meri finely serrated; anterior margins of carpi and propodi line with thick tomentum. G1 (Fig. 7I–L) elongated, relatively stout almost straight.

##### Distribution.

China (Zhejiang, Fujian), Taiwan (Kinmen), Vietnam (Khanh Hoa; Haiphong; Quang Ninh), and Malay Peninsula (including Singapore) (Fig. 1).

##### Remarks.

One curious record of *M. indica* was reported by Rathbun (1931) from Tsimei, Amoy (= Jimei, Xiamen), along the coasts of Fujian. Other than this record, there have been no definite records of *M. indica* from China, despite that of Shen (1940a) citing that of Rathbun’s (1931). Given the considerable resemblance between *M. indica* (see Naderloo 2011: figs 15, 18c–d; Naderloo 2017: figs 31.11e, 32.2, 32.3) and *M. sheni*, and the latter described from the region (Amoy) shortly before, it is reasonable to consider Rathbun’s (1931) record represented *M. sheni*.

Specimens collected from Kinmen, opposite Xiamen (Fujian, China), are confirmed as *M. sheni* based on molecular analyses (see below), being a new record to Taiwan.

#### Metaplax tredecim

Taxon classificationAnimaliaDecapodaVarunidae

Tweedie, 1950

676E9A4F-051C-525E-930C-93CA87289996

**Figure 6.**
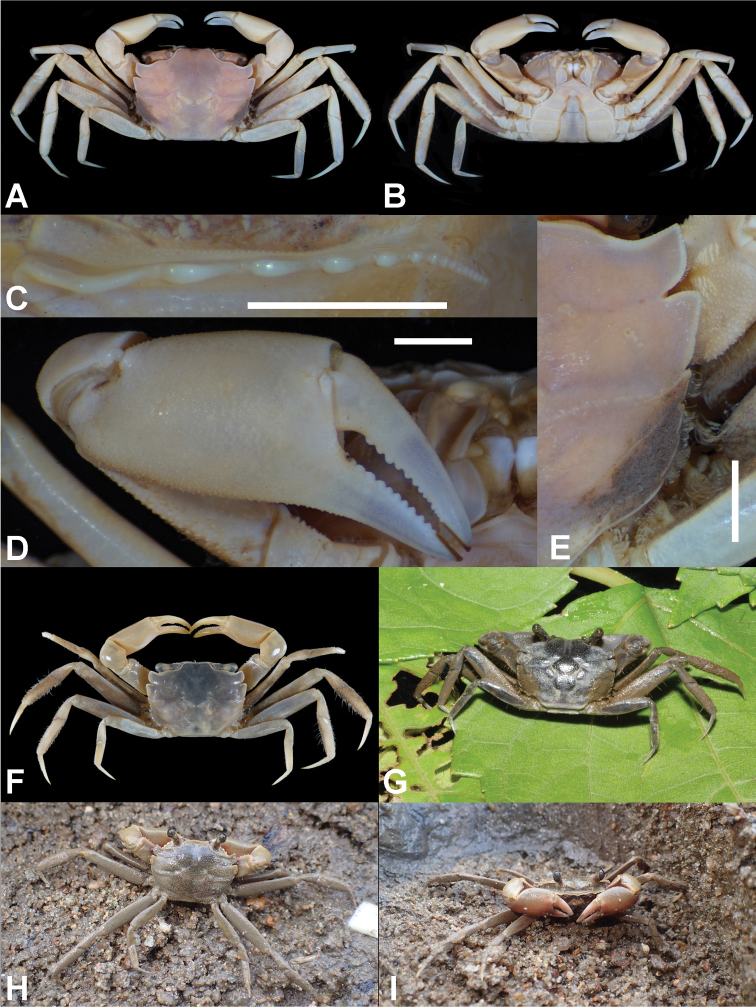
*Metaplax tredecim* Tweedie, 1950. **A, F** Dorsal view **B** ventral view **C** left infraorbital ridge **D** right cheliped **E** right side of carapace showing the five teeth of lateral margin. **A–E** paratype male (CW 16.2 mm; ZRC 1964.7.14.4-18; Labuan) **F** male (CW 16.5 mm; NCHUZOOL 15473; Hong Kong) **G** male (CW 15.4 mm; NCHUZOOL 15475; Labuan, Malaysia) **H, I** male (18.2 mm; NCHUZOOL 15705; Hong Kong) **G–I** color in life. Scale bars: 2.0 mm.

**Figure 7.**
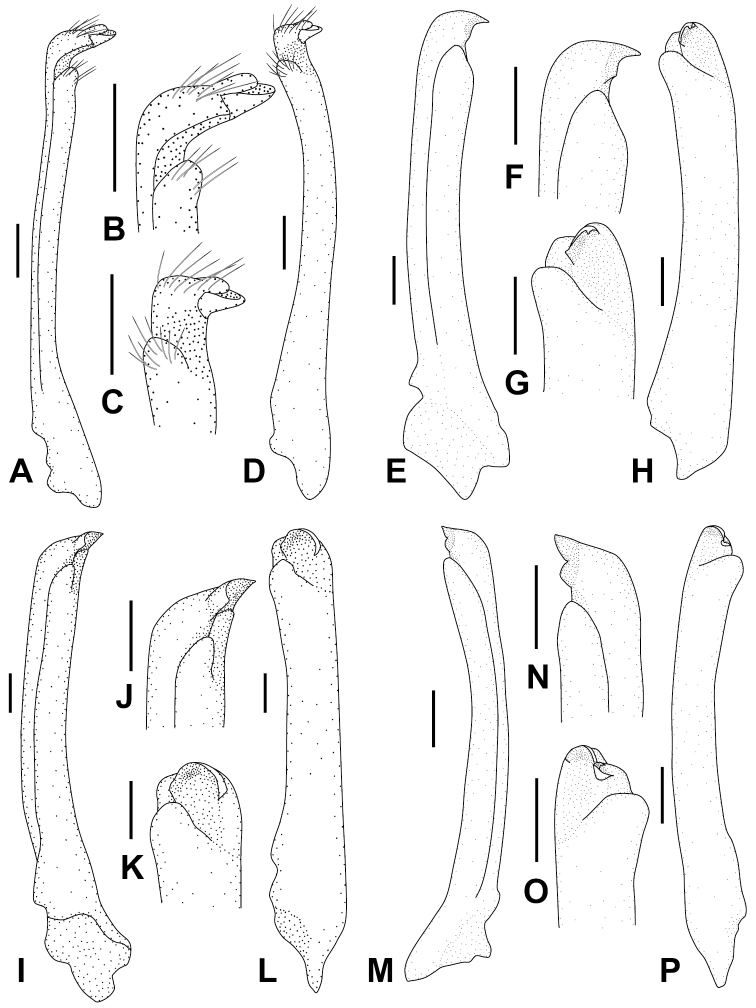
*Metaplax elegans* De Man, 1888 (**A–D**NCHUZOOL 15496, male, 12.7 mm), right G1; *M. longipes* Stimpson, 1858 (**E–H**ZRC 2019.0581, male, 14.9 mm), right G1; *M. sheni* Gordon, 1930 (**I–L**NCHUZOOL 15466, male, 9.9 mm), right G1; and *M. tredecim* Tweedie, 1950 (**M–P** paratype, ZRC 1964.7.14.4-18, 16.2 mm), left G1. Scale bars: 0.5 mm (**A–H, M–P**); 0.2 mm (**I–L**).

Figures 2J–L , 6 , 7M–P

Metaplax tredecim Tweedie, 1950: 354, fig. 6 (type locality: Labuan, Malaysia); Choy and Booth 1994: 243 (Brunei); Davie and Nguyen 2003: 383, fig. 1d–e (Malaysia: Labuan; Brunei); Ng et al. 2008: 226 (list); Yang et al. 2008: 803 (list; East China and South China seas).
Metaplax longipes : Dai et al. 1986: 508, fig. 288 (1–2), pl. 72(3) (China: Zhejiang, Fujian, Guangdong) (part); Dai and Yang 1991: 556, fig. 288 (1–2), pl. 72(3) (China: Zhejiang, Fujian, Guangdong) (part); Chertoprud et al. 2012: 276, pl. 47F (Vietnam: Nha Phu, Nha Trang, Khanh Hoa) (not M. longipes Stimpson, 1858).

##### Materials examined.

**Paratypes**: 2 ♂♂ (15.6–16.2 mm), 1 ♀ (15.7 mm) (ZRC 1964.7.14.4-18), Labuan, Malaysia, coll. G. Nunong, Aug. 1938. **Others. Hong Kong**: 1 ♂ (14.2 mm) (NCHUZOOL 15468), Starfish Bay, coll. P.-C. Tsai and H. Y. Cheung, 19 July 2015; 3 ♂♂ (16.4–19.4 mm) (NCHUZOOL 15546), Starfish Bay, coll. K. J. H. Wong, 4 June 2019; 1 ♂ (16.1 mm) (NCHUZOOL 15469), Tai Tan, coll. K. J. H. Wong, 15 July 2015; 1 ♂ (18.1 mm) (NCHUZOOL 15470), Ting Kok, coll. K. J. H. Wong, 22 Aug. 2017; 2 ♂♂ (12.8–16.2 mm), 1 ♀ (15.6 mm) (NCHUZOOL 15471), Kei Ling Ha, coll. K. J. H. Wong, 31 Aug. 2011; 3 ♂♂ (16.9–21.0 mm) (NCHUZOOL 15472), Nai Chung, coll. K. J. H. Wong, 23 June 2015; 2 ♂♂ (16.5–21.5 mm) (NCHUZOOL 15473), Luk Keng, coll. C. W. Lau, 22 May 2016; 1 ♂ (18.2 mm) (NCHUZOOL 15705), Mak Pin, Sai Kung, coll. K. J. H. Wong, 7 July 2019. **China**: 4 ♂♂ (18.0–22.7 mm), 1 ♀ (10.7 mm) (NCHUZOOL 15474), Dongzhai Harbor, Hainan, 23 June 2004. **Vietnam**: Quang Ninh: 1 ♂ (21.8 mm), 2 ♀♀ (15.7–23.4 mm) (NCHUZOOL 15476), Dong Rui, 29 May 2016; 1 ♂ (22.4 mm) (NCHUZOOL 15477), Dong Rui, coll. H.-T. Shih and P.-Y. Hsu, 9 Oct. 2017; 1 ♀ (10.3 mm) (NCHUZOOL 15478), Dong Rui, coll. H.-T. Shih and P.-Y. Hsu, 9 Oct. 2017; Khanh Hoa: 4 ♂♂ (14.4–18.7 mm), 5 ♀♀ (12.6–16.1 mm) (NCHUZOOL 15498), Nha Trang, coll. I.-H. Chen and K. J. H. Wong, 24 Nov. 2010. **Malaysia**: Labuan, 1 ♂ (15.4 mm), 1 ♀ (16.2 mm) (NCHUZOOL 15475), coll. H.-T. Shih, 23 July 2010; 1 ♂ (17.1 mm) (NCHUZOOL 15497), coll. H.-T. Shih, 27 July 2010.

##### Diagnosis.

Carapace (Figs 2J, 6A, F, G) subquadrate, 1.35 times broader than long (*N* = 39, SD = 0.03), regions defined by shallow grooves, slightly inflated, surface pitted; front nearly straight, medially slightly concave; lateral margin mildly convex, posteriorly converging, cut into five teeth, anterior two pronounced, quadrate, posterior two inconspicuous; posterolateral facet slightly depressed, behind second notch decorated with two short oblique granular ridges. Infraorbital ridge (Figs 2K, 6C) markedly sexually dimorphic: in males medial four or five roughly same size, decreasing in breadth laterally, laterally of a row of seven or eight small, rounded, isomorphic, tubercles; females with 21–27 small isomorphic tubercles. Chelipeds (Figs 2L, 6D) stout, symmetrical, palm 2.3 times longer than broad, length of palm approximately 1.6 times of length of dactyl (*N* = 12), meri slightly dilated anteriorly, lined with minute denticles along the margin; chela surface finely granulated, along cutting edges both fingers unarmed. Ambulatory legs slender, elongated, meri of P3 and P4 tomentum-covered on distal half, and propodi of P2 to P4 with thick mat of setae. G1 (Fig. 7M–P) elongated, slender, almost straight.

##### Distribution.

Southeast and East Asia: northern Borneo (Labuan, Malaysia; Brunei), Vietnam (Quang Ninh; Khanh Hoa), and South China (Hong Kong) (Fig. 1).

##### Habitat.

In Hong Kong, in comparison to *M. longipes*, *M. tredecim* tends to occur in habitats of coarser, grittier substrates, with less freshwater input, and frequently on open sandflats rather unsheltered by mangroves.

##### Remarks.

Identification of the *Metaplax tredecim* had been confusing, particularly based on the number of teeth along the lateral margin of the carapace. Tweedie (1950: fig. 6) showed merely three conspicuous lobes, the posterior one occupying more than half of carapace length. However, as noted by Davie and Nguyen (2003), members of the genus often have the structures around the posterolateral facet obscured by setae-trapped sediments, and not visible unless carefully denuded. Reexamination of a paratype male (16.2 mm; ZRC 1964.7.14.4-18), after denudation, showed the lateral margin to be interrupted by 4 notches (hence 5 teeth) (Figs 2J, 6A, E), the posterior two being inconspicuously defined by the last notch.

As mentioned above, two forms, differing in the number of tubercles or lobes on the infraorbital ridge, were recognized in specimens identified with *M. longipes* by Dai et al. (1986) and Dai and Yang (1991). We confirmed that specimens from the study area, characterized by the possession of about 17 tubercles or lobes closely represent *M. tredecim*. There is little doubt that the material studied by Dai et al. (1986) and Dai and Yang (1991) included two species, *M. longipes* and *M. tredecim*.

Chertoprud et al. (2012) recorded “*M. longipes*” from Nha Phu, southeastern Vietnam. However, given the infraorbital ridge with 17 tubercles and the chelipeds with the length of palm/the length of dactyl ratio about 1.5 (estimated from their plate 47F on page 295), this record is suspected to represent *M. tredecim* instead (see Table 2).

### Note on the number of infraorbital tubercles and lobes

**Figure 8.**
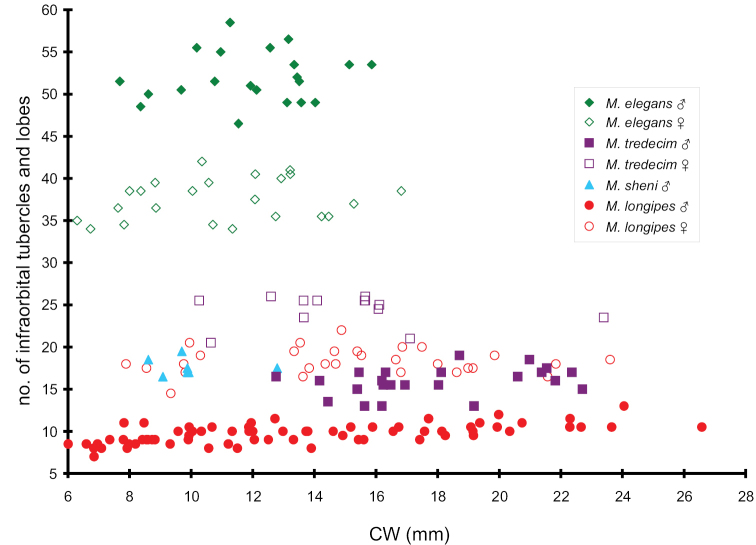
The number of infraorbital tubercles and lobes plotted as a function of carapace width (CW) of *Metaplax elegans* De Man, 1888, *M. longipes* Stimpson, 1858, *M. sheni* Gordon, 1930, and *M. tredecim* Tweedie, 1950.

As one of the major morphological features for the identification of species of *Metaplax* species, the number of lobes and granules along both infraorbital ridges, which are in all cases sexually dimorphic, differs substantially among species. The following range indicate the number of these lobes and tubercles of both sexes (with the exception of *M. sheni* for which only males were collected), with differences between left and right ridges placed in brackets: in *M. elegans* 46–61 (0–5) for males and 33–42 (0–3) for females, *M. longipes* 7–13 (0–2) for males and 14–22 (0–2) for females, *M. sheni* 16–20 (0–2) for males, and *M. tredecim* 13–20 (0–3) for males and 20–27 (0–3) for females (Table 2; Fig. 8). Tubercle counts overlap slightly in specimens of both sexes in *M. longipes* and *M. tredecim* (13 in males and 20–22 in females) and completely in male specimens of *M. sheni* (16–20) and *M. tredecim* (13–30). Comparing sexes, *M. longipes* and *M. tredecim* counts for males are less than females, but the reverse is true for *M. elegans*. These figures, however, do not show a clear trend in relation to body size (Table 2; Fig. 8).

### Molecular analyses

**Figure 9.**
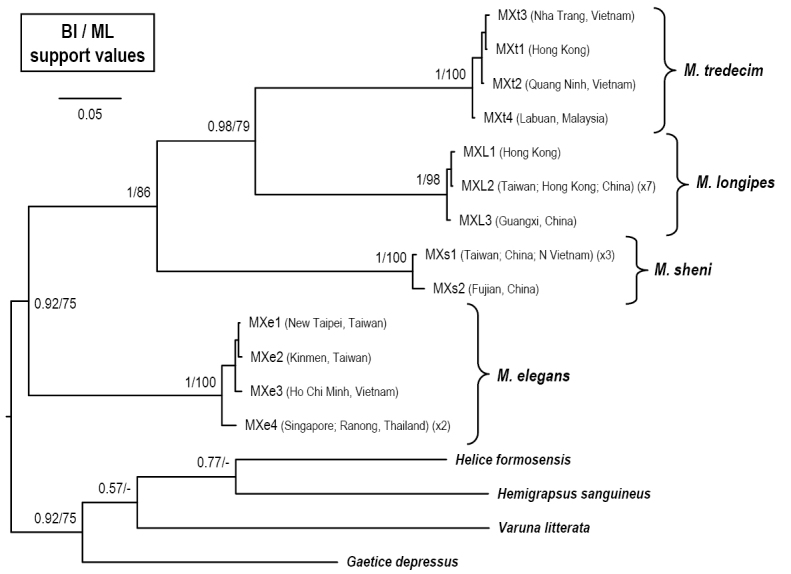
A Bayesian inference (BI) tree for *Metaplax elegans* De Man, 1888, *M. longipes* Stimpson, 1858, *M. sheni* Gordon, 1930, and *M. tredecim* Tweedie, 1950, and the outgroups, based on the cytochrome c oxidase subunit I (COI) gene. Probability values at the nodes represent support values for BI and maximum likelihood (ML). For haplotype names, see Table 1.

The molecular analysis of the COI marker included 22 specimens of *Metaplax*, with 13 haplotypes (Table 1). The phylogenetic reconstruction (Fig. 9) shows four well-supported clades, which could correspond to the four species treated in this study. It is obvious that only one clade is represented by specimens of *M. longipes* from South China and specimens from the type locality of *M. takahasii* (Danshuei, Taiwan). *Metaplax longipes* and *M. tredecim* are in sister-relation, and the two species and *M. sheni* form a main clade. *Metaplax elegans* is distant from other species of *Metaplax*.

The mean pairwise nucleotide divergence with K2P distances and bp differences of haplotypes of the four species are shown in Table 3. The intraspecific K2P nucleotide divergence of *M. elegans* (≤ 1.86 %) is higher than that of other species (≤ 0.92 %). The interspecific divergences among the four species are ≥ 15.87 %.

**Table 3.** Matrix of percentage pairwise nucleotide divergence with K2P distance (lower left) and mean number of differences (upper right) based on COI within and between species of *Metaplax* from East Asia and northern Vietnam (see Table 1). Values of range are shown in parentheses.

	Intraspecific		Interspecific			
	nucleotide divergence	Mean nucleotide difference	*M. elegans*	*M. longipes*	*M. sheni*	*M. tredecim*
*M. elegans*	1.21 (0–1.86)	7.8 (0–12)		106.47 (104–108)	102 (98–108)	110.35 (107–112)
*M. longipes*	0.1 (0–0.46)	0.67 (0–3)	18.36 (17.86–18.67)		102 (101–103)	97.75 (93–101)
*M. sheni*	0.46 (0–0.92)	3 (0–6)	17.5 (16.7–18.7)	17.5 (17.3–17.71)		104.44 (102–106)
*M. tredecim*	0.48 (0.15–0.92)	3.17 (1–6)	19.12 18.45–19.45)	16.8 (15.87–17.45)	17.99 (17.51–18.3)	

## Discussion

In this study, based on morphological and molecular evidences, we resolve the taxonomic confusions and updated the distribution of *Metaplax* species from East Asia and northern Vietnam. The presence of four species, viz., *M. elegans*, *M. longipes*, *M. sheni*, and *M. tredecim* are confirmed, and it is verified that *M. takahasii* is conspecific with *M. longipes*, and thus synonymized.

With regard to the number of infraorbital tubercles and lobes, despite elaborate sexual dimorphism among varunid species, serve as a reliable morphological feature in identifying *Metaplax* species (cf. Table 2), whereas the numbers of *M. elegans* substantially exceed those of congeners (Fig. 8). The numbers of infraorbital tubercles of the *Helice/Chasmagnathus* complex (Varunidae) are also used for species identification (K. Sakai et al. 2006), and likewise for species of *Helicana* K. Sakai & Yatsuzuka, 1980, all supported by genetic evidences. However, three species belong to the “*Helice latimera* complex” under *Helice* De Haan, 1833, with varying ranges of tubercle count, were shown to be otherwise (Shih and Suzuki 2008; Ng et al. 2018). The latter case implied *H. latimera* Parisi, 1918; *H. formosensis* Rathbun, 1931; and *H. tientsinensis* Rathbun, 1931 might well belong to a single species, as discussed in Ng et al. (2018). This ambiguity of specific delimitation requires further morphological and developmental investigations.

Phylogenetic relationships in the genus *Metaplax* or among genera of the Varunidae are far from settled. Monophyly of *Metaplax* has not yet been confirmed. Moreover, despite various recent research effort employing even complete mitochondrial sequences, the sister group of *Metaplax* remains unclear (Kitaura et al. 2002; Chen et al. 2019; Liu et al. 2019), which is probably due to the limited genera sampled in phylogenetic analyses.

In our study, the four species of *Metaplax* can be separated by the COI marker with a minimum interspecific distance of 17.5 %, which is higher than that of most other crab species (see Chu et al. 2015). The phylogenetic tree based on COI (Fig. 9) showed *M. longipes* and *M. tredecim* as sister species, and both species form a clade that is sister to *M. sheni*, whereas *M. elegans* is sister to the three as a whole. The phylogenetic relationships of the four species are also consistent with the number of infraorbital tubercles and lobes, i.e., *M. longipes* and *M. tredecim*, have the fewest number of these structures; *M. sheni* has moderate number; and *M. elegans* has the greatest number (Table 2; Fig. 8). This implies the number of infraorbital tubercles is possibly higher in the ancestral form, becoming reduced in successive clades. More species of this genus, however, should be included in the future to test this hypothesis.

Species of *Metaplax* are mainly distributed in the tropical and subtropical continental regions, in muddy and muddy sand habitats, always accompanied by mangroves (Sakai 1939, 1976; Dai et al. 1986; Dai and Yang 1991). It has been suggested that the pattern of geographical distributions agrees with the “continental type” of fiddler crabs, in contrast with the “oceanic type” mainly inhabited on islands (cf. Shih et al. 2010, 2016; Shih 2012). This also explains why no East Asian species of *Metaplax* are recorded from Korea, the main islands of Japan, the Ryukyu islands, and eastern Taiwan. The habitat preferred by species of *Metaplax* is suggested to be related to physiological constraints (e.g., food, temperature, salinity, etc.; Curtis and McGaw 2012; Theuerkauff et al. 2018). Understanding of the population structure may help reveal larval dispersal in the region of East Asia and northern South China Sea (Chan et al. 2007; Shih et al. 2015; Wang et al. 2015; Chai et al. 2017).

The results of this study clarify the biogeographic distribution of three species (Fig. 1). With *M. takahasii* synonymized with *M. longipes*, the distribution of *M. longipes* stretches from western Taiwan (including Matsu and Kinmen) to China (from Jiangsu to Guangxi) and northern Vietnam. *Metaplax sheni* is found in China (Zhejiang and Fujian), Taiwan (Kinmen), Vietnam, and Malay Peninsula (including Singapore); and the known range of *M. tredecim* include South China (Hainan and Hong Kong), Vietnam, and northern Borneo.

## Supplementary Material

XML Treatment for Metaplax elegans

XML Treatment for Metaplax longipes

XML Treatment for Metaplax sheni

XML Treatment for Metaplax tredecim
